# 276. SARS-CoV-2 Infection in Solid Organ Transplant Candidates and Recipients at Texas Children’s Hospital: A Retrospective Review

**DOI:** 10.1093/ofid/ofab466.478

**Published:** 2021-12-04

**Authors:** Leanne Petters, Flor M Munoz, Claire Bocchini, Elizabeth A Moulton, Daniel Ruderfer

**Affiliations:** 1 Baylor College of Medicine, Santa Fe, Texas; 2 Baylor College of Medicine and Texas Children’s Hospital, Houston, TX; 3 Baylor College of Medicine/Texas Children’s Hospital, Houston, Texas

## Abstract

**Background:**

Texas Children’s Hospital (TCH) is the largest pediatric solid organ transplant (SOT) program in the US, performing heart, kidney, liver, and lung transplants. Limited data exists about SARS-CoV-2 infection (COVID-19) in the pediatric SOT populations. We evaluated the impact of COVID-19 in a cohort of PCR positive SOT candidates and recipients. We hypothesized that COVID-19 would more severely impact SOT recipients compared to transplant candidates.

**Methods:**

Patients with SOT or transplant candidates at TCH with a positive SARS-CoV-2 RT-PCR test since March 1, 2020 to April 12, 2021 were included in the cohort. Retrospective medical record review was performed, and descriptive statistics were used.

**Results:**

A total of 103 SOT patients were identified, 13 candidates and 90 recipients. Of the SOT candidates, there were 1 heart, 3 kidney, and 9 liver transplant candidates. The SOT recipient cohort included 33 heart, 6 lung, 20 kidney, 33 liver and 2 multi-visceral recipients. A significant difference in age was observed between candidates and recipients with candidates being younger with median age of 4.5 years as opposed to recipient’s median age of 12.8 years (p=0.0003). The majority of patients, 70 of 101 (69%), were symptomatic. Most common symptoms reported were fever in 34/70 (49%), cough in 31/70 (44%), and headache in 19/70 (27%). A higher percentage of candidates (31%, 4 of 13) were hospitalized for acute COVID-19 infection compared to (17%, 15 of 90) of recipients. A transplant candidate who ultimately died from underlying illness and COVID-19 was the only patient in the cohort who required mechanical ventilation. More deaths (2/13, 15%) occurred in transplant candidates with COVID-19 compared to transplant recipients with COVID-19 (1/90, 1%, p=0.04); however, 2 of the deaths occurred after recovery from acute COVID-19 illness.

**Conclusion:**

Our study suggests that pediatric candidates who are actively listed for transplant with underlying conditions have more severe acute COVID-19 illness than pediatric SOT recipients despite their immunosuppression based on the higher mortality observed in the transplant candidates. Prospective studies are needed to better understand which specific patients are at increased risk for mortality from COVID-19.

**Disclosures:**

**Leanne Petters, MSN, APRN, CPNP-AC**, **Pfizer** (Scientific Research Study Investigator) **Flor M. Munoz, MD**, **Biocryst** (Scientific Research Study Investigator)**Gilead** (Scientific Research Study Investigator)**Meissa** (Other Financial or Material Support, DSMB)**Moderna** (Scientific Research Study Investigator, Other Financial or Material Support, DSMB)**Pfizer** (Scientific Research Study Investigator, Other Financial or Material Support, DSMB)**Virometix** (Other Financial or Material Support, DSMB) **Elizabeth A. Moulton, MD, PhD**, **Pfizer** (Scientific Research Study Investigator)

